# Mutation and Transmission Profiles of Second-Line Drug Resistance in Clinical Isolates of Drug-Resistant *Mycobacterium tuberculosis* From Hebei Province, China

**DOI:** 10.3389/fmicb.2019.01838

**Published:** 2019-08-07

**Authors:** Qianlin Li, Huixia Gao, Zhi Zhang, Yueyang Tian, Tengfei Liu, Yuling Wang, Jianhua Lu, Yuzhen Liu, Erhei Dai

**Affiliations:** ^1^Department of Epidemiology and Statistics, North China University of Science and Technology, Tangshan, China; ^2^Department of Laboratory Medicine, The Fifth Affiliated Hospital of Shijiazhuang, North China University of Science and Technology, Shijiazhuang, China

**Keywords:** second-line anti-tuberculosis drugs, *Mycobacterium tuberculosis*, associated-resistance mutation, acquired resistance, genotype

## Abstract

The emergence of drug-resistant tuberculosis (TB) is involved in ineffective treatment of TB, especially multidrug resistant/extensively resistant TB (MDR/XDR-TB), leading to acquired resistance and transmission of drug-resistant strains. Second-line drugs (SLD), including both fluoroquinolones and injectable drugs, were commonly proved to be the effective drugs for treatment of drug-resistant TB. The purpose of this study was to investigate the prevalence of SLD-resistant strains and its specific mutations in drug-resistant *Mycobacterium tuberculosis* clinical isolates, and to acknowledge the transmission pattern of SLD resistance strains in Hebei. The genes *gyrA*, *gyrB*, *rrs*, *eis* promoter and *tlyA* of 257 drug-resistant clinical isolates were sequenced to identify mutations that could be responsible for resistance against fluoroquinolones and second-line injectable drugs. Each isolate was genotyped by Spoligotyping and 15-loci MIRU-VNTR. Our results indicated that 48.2% isolates were resistant to at least one of five SLD. Of them, 37.7% isolates were resistant to fluoroquinolones and 24.5% isolates were resistant to second-line injectable drugs. Mutations in genes *gyrA*, *gyrB*, *rrs*, *eis* promoter and *tlyA* were detected in 73 (75.3%), 7 (7.2%), 24 (38.1%), 5 (7.9%), and 3 (4.8%) isolates, respectively. The most prevalent mutations were the D94G (23.7%) in *gyrA* gene and the A1401G (33.3%) in *rrs* gene. A combination of *gyrA*, *rrs* and *eis* promoter can act as a valuable predicator for predicting XDR phenotype. These results highlight the development of rapid diagnosis are the effective manners for the control of SLD-TB or XDR-TB.

## Introduction

Today, tuberculosis (TB) remains a major threat worldwide than any other single infectious disease. Millions of people continue to fall sick with TB each year. Moreover, the increasing rates of drug-resistant TB (DR-TB) worldwide and the emergence of multidrug/extensively-drug resistant TB (MDR/XDR-TB), leading to a high mortality as well as the financial burden. Regarding cases, estimates of the burden of DR-TB have focused on MDR-TB, there were 457,560 people (range: 396,060–523,980) developed MDR-TB (World Health Organization [WHO], 2018). Among cases of MDR-TB, 8.5% (*95%CI*: 6.2–11.0) were estimated to have XDR-TB (World Health Organization [WHO], 2018).

MDR-TB patients ordinarily require 18 months of treatment with recommendatory second-line anti-TB drugs (SLD) that primarily include fluoroquinolones (FQ) and second-line injectable drugs (SLID). Similar to other anti-TB agents, the main mechanisms of *Mycobacterium tuberculosis* (*M. tuberculosis*) resistant to SLD rely on spontaneous chromosomal mutations. Associated-mutations in the fluroquinolone resistance-determining region (QRDR) of DNA gyrase-coding genes *gyrA* and *gyrB* turned out to be responsible for FQ resistance (FQ^r^) (Chien et al., 2016). The genetic determinants of SLID resistance (SLID^r^) are more complicated and suggested that being involved in three well-known genes. Cross-resistance between kanamycin (KAN), amikacin (AMK) and capreomycin (CAP) is thought to be associated with mutations in the16S rRNA gene *rrs*, specifically at position A1401G (Alangaden et al., 1998; Oudghiri et al., 2018). Mutations in promoter region of *eis* gene, encoding an aminoglycoside acetyltransferase, caused low-level KAN resistance (Bauskenieks et al., 2015; Ngo et al., 2018). CAP resistance has been correlated with mutations in *tlyA* gene, which encodes a putative 2′-*O*-methyltransferase (TlyA) (Freihofer et al., 2016; Witek et al., 2017).

Currently, little is known about mutation profiles of SLD resistance (SLD^r^) in clinical *M. tuberculosis* isolates in our area, the objectives of this study were to compare the sequencing data of SLD^r^ associated-genes (FQ for *gyrA* and *gyrB*, SLID for *rrs* region 1400, *eis* promoter and *tlyA*) with the phenotypic results by the traditional proportion method in 275 drug-resistant clinical *M. tuberculosis* isolates collected in Hebei, and to analyze the *M. tuberculosis* genetic diversity of SLD^r^-TB (covering FQ^r^-TB and SLID^r^-TB), and to elucidate transmission pattern by using Spoligotyping and 15-loci MIRU-VNTR typing.

## Materials and Methods

### *M. tuberculosis* Isolates

**FIGURE 1:**
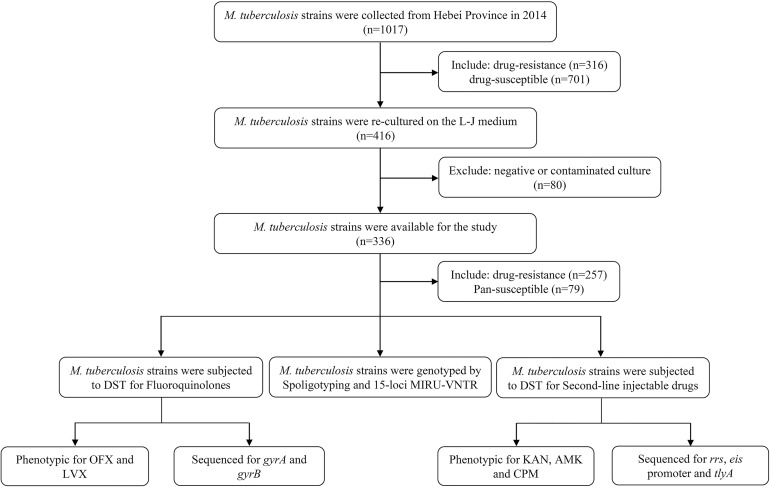
Flow chart and strains selection in this study.

For the present study, we successfully recovered 257 clinical *M. tuberculosis* isolates. Isolates were obtained from sputum samples provided by confirmed pulmonary patients (167 males and 90 females; age range: 14–83 years; median age: 41 years; 146 new patients and 111 retreated patients) attending eight hospitals in Hebei over 1-year period (From January to December in 2014). One hundred pan-susceptible *M. tuberculosis* isolates served as negative control. Figure 1 shows the selection process of clinical *M. tuberculosis* isolates originated from Hebei Province.

### Drug Susceptibility Testing

Drug susceptibility testing against SLD was performed using conventional proportion method on Lowenstein-Jensen (L-J) medium (Celnovte-bio, Henan, China) cultured for 4 weeks (World Health Organization [WHO], 1997), with the following break-out values: ofloxacin (OFX) 2 μg/mL; levofloxacin (LVX) 2 μg/mL; KAN 30 μg/mL; AMK 30 μg/mL, and CAP 40 μg/mL. Each DST was used *M. tuberculosis* H37Rv (ATC27294) as a reference control.

### DNA Isolation, Amplification, and Sequencing

Genomic DNA extractions were re-suspended a loopful of bacilli in 200 uL of fast lysis buffer (Qiagen, Valencia, CA, United States) and heat inactivated at 80°C for 10 min, followed by centrifugation at 13,000 *g* for 5 min. All genes were chosen on the basis of documented association with resistance to SLD and included *gyrA* and *gyrB* for FQ and *rrs*, *eis* promoter, and *tlyA* for SLID. Primers and amplicon sizes are presented in the Supplementary Table S1. Each 20 μL PCR mixture was prepared as follows: 10 μL 2× Taq Master Mix (CWBOIO, Beijing, China), 1 μL of the forward and reverse 10 μM primers, 1 μL genomic DNA, and final 7 μL distilled H_2_O complement. PCR program for amplification were 5 min at 94°C, followed by 35 cycles of 30 s at 94°C, 30 s at 60°C, 30 s at 72°C, and a final extension 72°C for 10 min.

Sequencing services were provided by Tsingke Biological Technology company (TsingKe, Beijing, China). All the mutations were identified and aligned with the homologous sequences of the reference *M. tuberculosis* H37Rv strain (GenBank accession number NC_000962) using the BLASTN algorithm^1^.

### Genotyping

Spoligotyping was used to identify Beijing family as described previously by Kamerbeek et al. (1997). The direct repeat region was amplified with the primer pairs included DRa (5′-GGTTTTGGGTCTGACGAC-3′) and DRb (5′-CCGAGAGGGGACGGAAAC-3′). All PCR products were hybridized to a set of 43 oligonucleotide probes corresponding to each spacer that were covalently bound to a membrane. Spoligotypes in binary formats were matched with Spoldb.4.0 database^2^, and applied the published rules for definition of Beijing family (hybridization to at least three of the spacers 35–43 in direct repeat region and absence of hybridization to spacers 1–34).

MIRU-VNTR typing method was based on 15-loci set, including Mtub04, ETRC, ETRD, MIRU40, MIRU10, MIRU16, Mtub21, QUB11b, ETRA, Mtub30, MIRU26, ETRE, Mtub39, QUB26, QUB4156, as previously reported (Supply et al., 2001; Li et al., 2016). A phylogenetic tree was built using UPGMA algorithm at the web site MIRU-VNTR*plus*^3^.

### Definitions

Based on the following definitions, we utilize these terms throughout the rest of this article. (i) Drug-resistant (DR): defined as resistance to at least one first- or second-line drug; (ii) Fluoroquinolones (FQ): defined as resistance to at least one OFX or LVX; (iii) Second-line injectable drugs (SLID): defined as resistance to at least one AMK, KAN, and CAP; (iv) Second-line anti-TB drugs (SLD): defined as resistance to at least one fluoroquinolones or second-line injectable drugs; (v) Multidrug-resistant (MDR): defined as resistance to at least two of the most effective anti-TB drugs, isoniazid and rifampin (World Health Organization [WHO], 2018); (vi) Pre-extensively drug-resistant (Pre-XDR): defined as MDR resistance to either fluoroquinolones or second-line injectable drugs; (vii) Extensively drug-resistant (XDR): defined as MDR-TB plus resistance to a fluoroquinolone and at least one second-line injectable drug (World Health Organization [WHO], 2018).

### Data Analysis

To assess the associations between variables (DST, mutations, and genotyping), the χ^2^ test, odds ratio (*OR*) and 95% confidence interval (*95%CI*) were calculated. And the Fishers’ exact was used if any expected counts are less than 5. A *P*-value of < 0.05 was considered statistically significant. All statistical data analyzed with SPSS version 22.0 (IBM SPSS, Chicago, IL, United States).

### Resolution of Discrepant Results

When the results between DST and DNA sequencing were not consistent, two methods were used for repeated testing. If the duplicate result conflicted with the original data, a third round of testing was accepted as a final value.

## Results

### Strains Selection and Drug Resistance Patterns

Initially, a total of 316 DR isolates were re-cultured and finally only 257 eligible isolates were included in this study. The selection procedure of strains is shown in Figure 1. A total 257 eligible isolates, 170 (170/257, 66.1%) isolates were resistant to isoniazid; 152 (152/257, 59.1%) to rifampicin; 74 (74/257, 28.8%) to ethambutol; and 176 (176/257, 56.8%) to streptomycin.

Among 257 DR isolates, 48.2% (124/257) were resistant to at least one of five SLD and the remaining 51.8% (133/257) were susceptible to all SLD. Of them, 37.7% (97/257) were FQ^r^ isolates and 24.5% (63/257) were SLID^r^ isolates. A total of 118 isolates (45.9%, 118/257) identified as MDR, of which 46 isolates (17.9%, 46/257) were Pre-XDR and 10.9% (28/257) were XDR. At the same time, there were 32.7% (84/257), 21.7% (56/257), 20.2% (52/257), 7.0% (18/257), and 6.2% (16/257) of 257 DR isolates were found to be resistant to OFX, LVX, KAN, AMK and CAP, respectively. Detailed profiles of 275 DR isolates against five SLD were summarized in Table 1.

**TABLE 1:** Profiles of drug resistance to second-line drugs among the studied drug-resistant isolates.

**DST patterns^a^**	**No. of isolates**	**Proportion (%)**
Mono-resistant		
OFX	24	9.34
LVX	9	3.5
KAN	20	7.78
AMK	1	0.39
CAP	2	0.78
Multi-resistant		
OFX + LVX	28	10.89
OFX + KAN	8	3.11
OFX + AMK	3	1.17
OFX + CAP	3	1.17
LVX + CAP	1	0.39
KAN + AMK	2	0.78
KAN + CAP	1	0.39
OFX + LVX + KAN	8	3.11
OFX + KAN + AMK	1	0.39
OFX + KAN + CAP	1	0.39
OFX + AMK + CAP	1	0.39
LVX + KAN + AMK	2	0.78
KAN + AMP + CAP	1	0.39
OFX + LVX + KAN + AMK	2	0.78
OFX + LVX + KAN + CAP	1	0.39
LVX + KAN + CAP + AMK	1	0.39
OFX + LVX + KAN + AMK + CAP	4	1.56
Susceptible to second-line drugs	133	51.8

*^*a*^DST, drug susceptibility testing; OFX, ofloxacin; LVX, levofloxacin; KAN, kanamycin; AMK, amikacin; CAP, capreomycin.*

**FIGURE 2:**
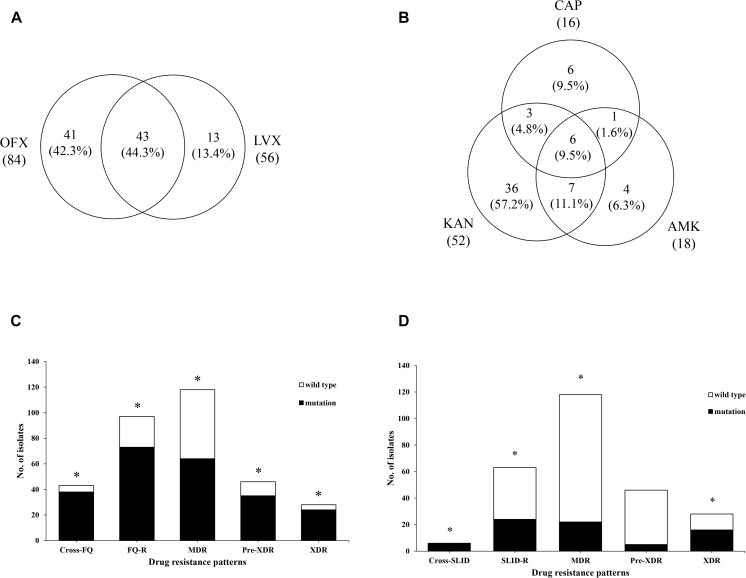
Analysis of phenotypic and genetic characterizations in different drug-resistance patterns. **(A)** Venn diagram of isolates identified from FQ group. The left circle shows any resistance to OFX, the right circle shows any resistance to LVX, the overlapping region shows isolates resistant to both OFX and LVX. **(B)** Venn diagram of isolates identified from SLID group. Each circles show any resistance to KAN, AMK, and CAP, respectively. The overlapping regions show isolates cross-resistant to the corresponding drugs. **(C)** The *gyrA* mutations were significantly associated with cross-resistance of FQ (4.13 *OR*, *95%CI* [1.39, 12.23], *P* = 0.009), resistance of FQ (45.63 OR, 95%CI [20.73,100.42], *P* < 0.001), MDR (7.49 *OR*, *95%CI* [4.09, 13.70], *P* < 0.001), Pre-XDR (10.81*OR*, *95%CI* [5.10, 22.88], *P* < 0.001), and XDR (17.29 *OR*, *95%CI* [5.76, 51.89], *P* < 0.001). **(D)** The *rrs* mutations were significantly associated with cross-resistance of SLID (3.17 *OR*, *95%CI* [2.16, 4.64], *P* = 0.002), resistance of SLID (9.23 *OR*, *95%CI* [9.60, 88.98], *P* < 0.001), MDR (5.08 *OR*, *95%CI* [1.98, 13.00], *P* < 0.001), and XDR (24.11 *OR*, *95%CI* [9.35, 62.20], *P* < 0.001). ^*^Indicates the difference is statistically significant (*P* < 0.05).

**FIGURE 3:**
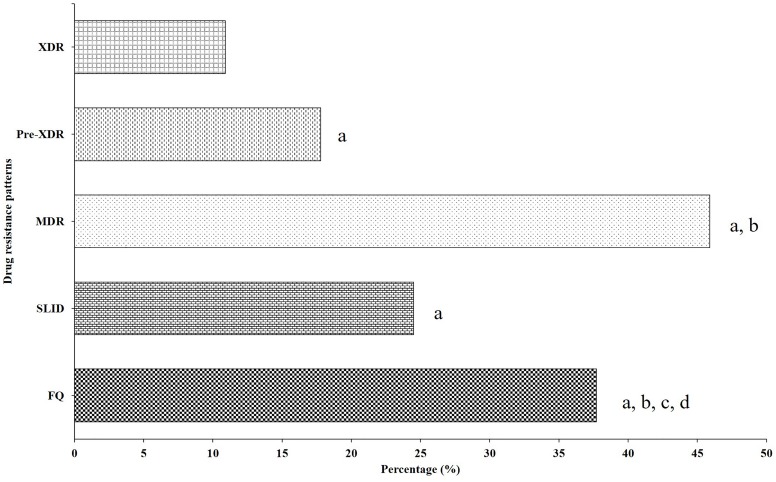
Distribution of drug-resistant strains resistant to different second-line drug group. ^a^Compared with XDR group, there was a significantly lower than FQ (0.20 *OR*, *95%CI* [0.13, 0.32], *P* < 0.001), SLID (0.38 *OR*, *95%CI* [0.23, 0.61], *P* < 0.001), Pre-XDR (0.56 *OR*, *95%CI* [0.34, 0.93], *P* = 0.024) and MDR (0.14 *OR*, *95%CI* [0.09, 0.23], *P* < 0.001). ^b^Compared with Pre-XDR group, there was significantly lower than FQ (0.36 *OR*, *95%CI* [0.24, 0.54], *P* < 0.001), and MDR (0.26 *OR*, *95%CI* [0.17, 0.38], *P* < 0.001). ^c^Compared with MDR group, there was significantly higher than FQ (2.61 *OR*, *95%CI* [1.80, 3.81], *P* < 0.001). ^d^Compared with SLID group, there was significantly lower than FQ (1.87 *OR*, *95%CI* [1.27, 2.73], *P* = 0.001).

Out of 84 OFX^r^ isolates, 43 isolates (44.3%, 43/97) were simultaneously resistant to LVX (Figure 2A). Among 52 KAN^r^ isolates, 6 isolates were resistant to both AMK and CAP, the cross-resistance rate between KAN, AMK and CAP was 9.5% (6/64) (Figure 2B). Compared with FQ^r^, there was significant difference in SLID^r^ (37.7 vs. 24.5%, 1.9 *OR*, *95%CI* [1.3, 2.7], *P* = 0.001), MDR (37.7 vs. 45.9%, 2.6 *OR*, *95%CI* [1.8, 3.8], *P* < 0.001), Pre-XDR (37.7 vs. 17.9%, 0.4 *OR*, *95%CI* [0.2, 0.5], *P* < 0.001) and XDR (37.7 vs. 10.9%, 0.2 *OR*, *95%CI* [0.1, 0.3], *P* < 0.001) (Figure 3). Detailed distribution of drug-resistant strains resistant to OFX, LVX, KAN, AMK and CAP as shown in Supplementary Figure S1.

### Mutations in *gyrA* and *gyrB*

It is known to that the main molecular mechanism of FQ^r^ was caused by mutations in the QRDR of DNA gyrase, which composed of GyrA and GyrB subunits, encoded by *gyrA* and *gyrB* genes, respectively. DNA sequencing results of these two genes from FQ^r^, FQ^s^ and pan-susceptible isolates were summarized in Table 2.

**TABLE 2:** Analysis of the *gyrA* and *gyrB* mutations in resistant, sensitive to FQ and to pan-susceptible *M. tuberculosis* isolates.

**Fluoroquinolones^a^**		***gyrA*^b^**		***gyrB***		**No. of isolates**
			
**OFX**	**LVX**	**Codon change(s)**	**Amino acid change(s)**	**Codon change(s)**	**Amino acid change(s)**	
R	R	GCG269GTG	A90V	WT	WT	9
		GCG269GTG	A90V	AAC1496ACC	N499T	1
		GAC281GGC	D94G	WT	WT	13
		GAC281GCC	D94A	WT	WT	3
		GAC280TAC	D94Y	WT	WT	3
		GAC280TAC	D94Y	GCG1534GCA	A508A	1
		GAC280AAC	D94N	WT	WT	7
		GAC265AAC	D89N	WT	WT	1
		WT	WT	GAC1381AAC	D461N	1
R	S	GCG269GTG	A90V	WT	WT	7
		GAC281GGC	D94G	WT	WT	7
		GAC281GGC	D94G	CTG1324TTG	L442L	1
		GAC281GCC	D94A	WT	WT	3
		GAC280TAC	D94Y	WT	WT	2
		GAC280AAC	D94N	WT	WT	4
		WT	WT	GCG1511GTG	A504V	1
		WT	WT	TCC1340TTC	S447F	1
		WT	WT	GCG1534GCA	A508A	1
S	R	TCG271CCG	S91P	WT	WT	2
		GCG269GTG	A90V	WT	WT	5
		GAC281GGC	D94G	WT	WT	2
		GAC280TAC	D94Y	WT	WT	2
S	S	GCG269GTG	A90V	WT	WT	2
		GAC281GGC	D94G	WT	WT	2
		GAC281GCC	D94A	WT	WT	5
		CGC318CGA	R106R	WT	WT	1
		WT	WT	GCG1534GCA	A508A	5
		WT	WT	AAG1322AGG	K441R	1
		WT	WT	TCC1340TAC	S447Y	1
Pan-susceptible		GCG269GTG	A90V	WT	WT	2
		GAC281GGC	D94G	WT	WT	1
		GAC281GCC	D94A	WT	WT	1
		WT	WT	GGT1307GTT	G436V	1

*^*a*^OFX, ofloxacin; LVX, levofloxacin; R, resistance; S, sensitivity. ^*b*^WT, wild-type.*

Of 97 FQ^r^ isolates, 73 isolates (73/97, 75.3%) carried mutations within *gyrA* gene, including codons 89, 90, 91, and 94. The most predominant mutation occurred at codon 94, with four different amino acid changes, D94G (23/97, 23.7%), D94A (6/97, 6.2%), D94Y (8/97, 8.2%), and D94N (11/97, 11.3%), which accounted for 49.5% (48/97) of FQ^r^ isolates. The A90V was the next most prevalent mutation, 22.7% (22/97) of isolates harbored this mutation. Besides, a single mutation in or near the QRDR of *gyrB* gene associated with FQ^r^ was detected in 4 isolates, including D461N (1/97, 1.0%), A504V (1/97, 1.0%), S447F (1/97, 1.0%) and A508A (1/97, 1.0%). Double mutations in both *gyrA* and *gyrB* were observed in three isolates, one with A90V and N499T mutation, one with D94Y and A508A mutation and one with D94G and L442L.

Of 160 FQ^s^ isolates, 17 isolates (17/160, 10.6%) displayed mutations within these two target fragments (*gyrA* and *gyrB*). Among them, the mutations within *gyrA* included A90V (2/160, 1.3%), D94G (2/160, 1.3%), D94A (5/160, 3.1%), and R106R (1/160, 0.6%) and mutations within *gyrB* were observed in 7 isolates with A508A (5/160, 3.1%), K441R (1/160, 0.6%), and S447Y (1/160, 0.6%).

Among 79 pan-susceptible isolates, 3 isolates each had mutations at codon 90 (A-V),94 (D-G), or 94 (D-A) in *gyrA* gene. Mutation at codon 436 (G-V) of *gyrB* gene was observed only one isolate.

### Mutations in *rrs, eis* Promoter and *tlyA*

To determine the molecular basis of resistance to SLID, the *rrs*, *eis* promoter, and *tlyA* region were sequenced both in SLID^r^, SLID^s^, and pan-susceptible strains. Table 3 showed the mutations in the 1400 region of *rrs*, *eis* promoter and *tlyA* as well as the corresponding resistance phenotypes.

**TABLE 3:** Analysis of *rrs*, *eis* promoter and *tlyA* mutations in resistant, sensitive to SLID and to pan-susceptible *M. tuberculosis* isolates.

**Second-line injectable drugs^a^**			***rrs*^b^**	***eis* promoter**	***tlyA***		**No. of isolates**
				
**KAN**	**AMK**	**CAP**	**Nucleotide change(s)**	**Nucleotide change(s)**	**Codon change(s)**	**Amino acid change(s)**	
R	R	R	A1401G	WT	WT	WT	6
R	R	S	A1401G	WT	WT	WT	3
			WT	G (−10) A	WT	WT	1
R	S	R	A1401G	WT	WT	WT	1
			WT	G (−10) A	WT	WT	1
R	S	S	A1401G	WT	WT	WT	8
			T1491C	WT	WT	WT	1
			A1499G	WT	WT	WT	1
			WT	G (−10) A	WT	WT	1
			WT	C (−14) T	WT	WT	2
S	R	S	A1401G	WT	WT	WT	2
			G1454A	WT	WT	WT	1
S	S	R	A1401G	WT	WT	WT	1
			WT	WT	AAA205GAA	K69E	1
			WT	WT	GCG356GAG	A119E	1
			WT	WT	AAA567AAC	K189N	1
S	S	S	A1128G	WT	WT	WT	1
			A1138G	WT	WT	WT	1
			C1209T	WT	WT	WT	1
			C1483T	WT	WT	WT	1
Pan-susceptible			WT	G (−37) T	WT	WT	1
			WT	WT	GTG161GGG	V54G	1
			WT	WT	ACC159ACT	T53T	1

*^*a*^KAN, kanamycin; AMK, amikacin; CAP, capreomycin; R, resistance; S, sensitivity. ^*b*^WT, wild-type.*

Of 63 SLID^r^ isolates, the most frequent mutation was the change from A to G at position 1401 of *rrs* gene, observed in 33.3% (21/63) of isolates. Three additional isolates displayed mutations at T1491C, G1454A, and A1499G in *rrs*. Mutations within the promoter region of *eis* included G (−10) A (3/63, 4.8%) and C (−14) T (2/63, 3.2%). Nevertheless, mutations in *tlyA* conferring amino acid substitutions K69E (1/63, 1.6%), A119E (1/63, 1.6%), and K189N (1/63, 1.6%), solely appeared in CAP^r^ isolates.

Of 193 SLID^s^ isolates, one isolate each of A1128G, A1138G, C1209T, and C1483T, all occurred in *rrs*. Furthermore, for *eis* promoter, a change from G to T at −37 was detected only one isolate.

Among 79 pan-susceptible isolates, none of mutations were tested by us, except that, two clinical isolates with mutations V54G and T53T in *tlyA*.

### Association Between Gene Mutations and Phenotypes

Among the 97 FQ^r^ isolates with *gyrA* mutations, 63.9% (62/97) were resistant to OFX and 50.5% (49/97) were resistant to LVX. As seen in Figure 2C, strongly evidence showed that *gyrA* mutations were associated with the cross-resistance of FQ (4.1 *OR*, *95%CI* [1.4, 12.2], *P* = 0.009), FQ (45.6 *OR*, *95%CI* [20.7, 100.4], *P* < 0.001), MDR (7.5 *OR*, *95%CI* [4.1, 13.7], *P* < 0.001), Pre-XDR (10.8 *OR*, *95%CI* [5.1, 22.9], *P* < 0.001), and XDR (17.3 *OR*, *95%CI* [5.8, 51.9], *P* < 0.001). On the contrary, there was no significant correlation between FQ^r^ isolates with *gyrB* mutations (1.7 *OR*, *95%CI* [0.6, 5.0], *P* = 0.331) (Table 4).

**TABLE 4:** Evaluation of phenotypic resistance of second-line anti-tuberculosis strains by mutations in second-line drug-resistant genes.

		**No. of isolates**						**Performance ^c^**			
					
**Drug^a^**	**Locus**	**Resistant**		**Sensitive**		***P*-value**	***OR* (*95%CI*)^b^**	**Accuracy value**		**Diagnostic**	
					
		**With mutation**	**Without mutation**	**With mutation**	**Without mutation**			**Sensitivity (%)**	**Specificity (%)**	**PPV (%)**	**NPV (%)**
FQ	*gyrA*	73	24	10	150	<0.001	46.0 (21.0,100.4)	75.2	94.0	88.0	86.2
	*gyrB*	7	90	7	153	<0.001	2.0 (1.0, 5.0)	7.2	96.0	50.0	63.0
	*gyrA, gyrB*	77	20	17	143	0.331	32.4 (16.0, 65.4)	79.4	89.4	82.0	88.0
SLID	*rrs*	24	39	4	190	<0.001	29.2 (10.0, 89.0)	38.1	98.0	86.0	83.0
	*eis* promoter	5	58	1	193	0.004	17.0 (2.0, 145.3)	8.0	99.5	83.3	77.0
	*tlyA*	3	60	0	194	0.014	1.0 (1.0, 1.0)	5.0	100.0	100.0	76.4
	*rrs*, *eis* promoter	29	34	5	189	<0.001	32.2 (12.0, 89.1)	46.0	97.4	85.3	85.0
	*eis* promoter, *tlyA*	8	55	1	193	<0.001	28.0 (3.4, 229.3)	13.0	99.5	89.0	78.0
	*rrs* and *tlyA*	27	36	4	190	<0.001	36.0 (12.0, 108.0)	43.0	98.0	87.1	84.1
	*rrs*, *eis* promoter, *tlyA*	32	31	5	189	<0.001	39.0 (14.1, 108.0)	51.0	97.4	86.5	86.0
Pre- XDR	*gyrA*	35	11	48	163	<0.001	11.0 (5.1, 23.0)	76.1	77.3	42.2	94.0
	*rrs*	5	41	23	188	>0.999	1.0 (0.4, 3.0)	11.0	89.1	18.0	82.1
	*gyrA, rrs*	37	9	55	156	<0.001	12.0 (5.3, 26.0)	80.4	74.0	40.2	95.0
XDR	*gyrA*, *rrs*, *eis* promoter	26	2	55	174	<0.001	41.1 (9.5, 179.0)	93.0	76.0	32.1	99.0

*^*a*^FQ, fluoroquinolones; SLID, second-line injectable drugs; Pre-XDR, pre-extensively drug-resistant; XDR, extensively drug-resistant. ^*b*^OR, odd ratio; CI, confidence interval. ^*c*^PPV, positive predictive value; NPV, negative predictive value.*

On the other side, we observed that 28.6% (18/63), 17.5% (11/63), and 12.7% (8/63) of resistant isolates with an A1401G mutation in *rrs* were resistant to KAN, AMK, and CAP, respectively. Also, we found *rrs* mutations were significantly associated with the cross-resistance of SLID (3.2 *OR*, *95%CI* [2.2, 4.6], *P* = 0.002), SLID (29.2 *OR*, *95%CI* [9.6, 89.0], *P* < 0.001), MDR (5.1 *OR*, *95%CI* [2.0, 13.0], *P* < 0.001), and XDR (24.1 *OR*, *95%CI* [9.4, 62.2], *P* < 0.001) (Figure 2D). Moreover, the isolates with *eis* promoter or *tlyA* gene mutations were related to the drug resistance to SLID (16.6 *OR*, *95%CI* [1.9, 145.3], *P* = 0.004; 1.0 *OR*, *95%CI* [0.9, 1.0], *P* = 0.014, respectively) (Table 4). Table 4 showed the sensitivity and specificity for evaluating phenotypes. For FQ^r^, mutations in *gyrA* and *gyrB* had a combined sensitivity of 79.4% (*95%CI* [69.7, 86.7]), and specificity of 89.4% (*95%CI* [83.3, 93.5]). For SLID^r^, *rrs* mutations were predicted phenotypes with 38.1% (*95%CI* [26.4, 51.2]) sensitivity, and 97.9% (*95%CI* [94.5, 99.3]) specificity. When adding the *eis* promoter and *tlyA* mutations, a combined sensitivity of 50.8% (*95%CI* [38.0, 63.5]), specificity of 97.4% (*95%CI* [93.8, 99.0]). The individual *gyrA* mutations for assessing Pre-XDR had sensitivity, and specificity values of 76.1% (*95%CI* [60.9, 86.9]), and 77.3% (*95%CI* [70.9, 82.6]), respectively, but adding the *rrs* mutations increased the values of sensitivity and for Pre-XDR detection to 80.4% (*95%CI* [65.6, 90.1]), and 73.9% (*95%CI* [67.4, 79.6]), respectively. A combined mutation of *gyrA*, *rrs* and *eis* promoter achieved the best evaluation for XDR with the sensitivity, and specificity values of 92.9% (*95%CI* [75.0, 98.8]), and 73.9% (*95%CI* [67.4, 79.6]), respectively.

### Genotyping

Three major diverse families were identified among the 124 SLD^r^ isolates, including Beijing, T and H family. Beijing family was the largest sub-lineage with 112 isolates. Nine isolates belonged to the T family, 6 isolates were T1 sublineage, 2 isolates were T2, and 1 isolate was T3. The remaining 3 isolates were identified as H3 sub-lineage.

Comparison of Spoligotyping results with three drug resistance patterns (Table 5), 116 isolates were grouped in 4 clusters and 8 isolates had singletons. Of the singletons, 7 isolates were distributed in clades, Beijing (SIT190, 260, and 621), T1 (SIT522 and 1688), T3 (Orphan) and H3 (SIT1908) with FQ-TB or SLID-TB. Only one Pre-XDR isolate was defined as T1 family (SIT520). The predominance of Beijing family (SIT1) contained 109 isolates clone in FQ-TB (71.8%, 89/124), SLID-TB (43.5%, 54/124) and Pre-XDR/XDR-TB group (56.5%, 70/124). Other three clusters, T1 (SIT53), T2 (SIT52) and H3 (SIT268), 2–3 isolates each, were found in Pre-XDR/XDR-TB and/or additional FQ or SLID. No significant associations were found between the genotypes and specific types of resistance (Supplementary Table S2). Meanwhile, no significant correlation was observed between any mutation and a Spoligotype family (Supplementary Table S3 and Supplementary Figure S2).

**TABLE 5:** Spoligotying characterization of FQ-TB, SLID-TB and Pre-XDR/XDR-TB.

**Clusters**	**No. of isolates**	**Spoligotype description octonary**	**SIT^a^**	**clades**	**FQ-TB^b^ (*n* = 97)**	**SLID-TB^c^ (*n* = 63)**	**Pre-XDR/XDR-TB^d^ (*n* = 74)**
Singleton	1	000000000003731	190	Beijing	1	0	0
	1	000000000003171	260	Beijing	1	0	0
	1	000000000002771	621	Beijing	1	1	0
	1	777777777760571	520	T1	1	0	1
	1	777777777760770	522	T1	0	1	0
	1	777777403760771	1688	T1	0	1	0
	1	577737777760771	Orphan	T3	1	1	0
	1	777777776020771	1908	H3	0	1	0
Cluster	109	000000000003771	1	Beijing	89	54	70
	3	777777777760771	53	T1	1	2	1
	2	777777777760731	52	T2	2	0	1
	2	777777677720771	268	H3	0	2	1

*^*a*^SIT, soligotyping international type. ^*b*^FQ-TB, fluoroquiolones-tuberculosis. ^*c*^SLID-TB, second-line injectable drugs. ^*d*^Pre-XDR, pre-extensively drug-resistant tuberculosis; XDR, extensively drug-resistant tuberculosis.*

**FIGURE 4:**
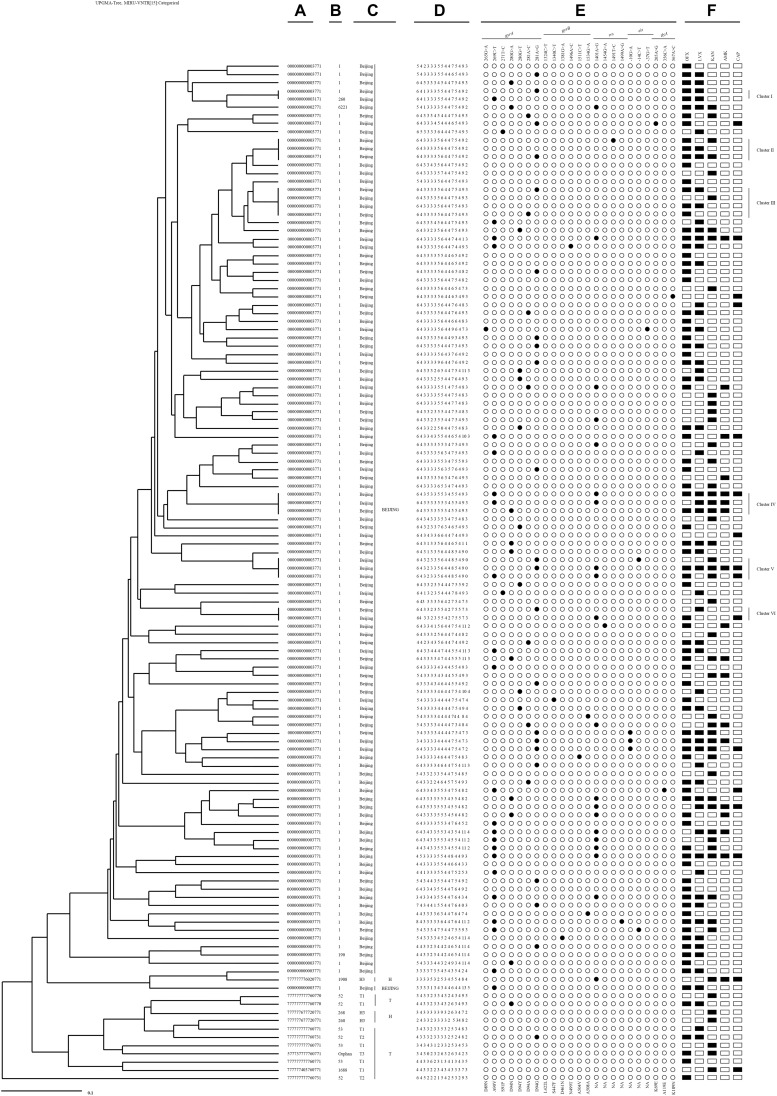
An UPGMA-tree based on 15-loci MIRU-VNTR of the clinical 124 *M. tuberculosis* isolates resistant to second-line drugs. From left to right: **(A)** Spoligotype octonary; **(B)** Spoligotype international type (SIT); **(C)** Spoligotype clade; **(D)** 15-loci MIRU-VNTR profile; **(E)** mutational pattern; **(F)** second-line drugs resistance pattern.

A total of 124 SLD^r^ isolates were genotyped by 15-loci MIRU-VNTR, revealed that the strains were divided into 113 genotypes, of which 107 were identified as unique patterns and 17 isolates were categorized to cluster patterns (Figure 4). Six small clusters ranging from 2 to 4 isolates were observed in all genotypes. The cumulative clustering rate and recent transmission rate rely on the MIRU-VNTR typing was 13.7% (17/124) and 4.8% (6/124), respectively.

Among six clusters with SLD^r^ strains, two Pre-XDR isolates with a *gyrA* (A90V or D94G) mutation occurred exclusively in Cluster I. Cluster II contained three isolates, two Pre-XDR isolates presented at *gyrA* mutation (D94G), one of them accompanied with a mutation at *rrs* (T1491C), the rest of FQ had no mutation. Cluster III grouped in 4 isolates, the Pre-XDR included 2 isolates with a mutation at *gyrA* (D94G or D94A), one was FQ and another was SLID without any mutation. Three isolates of cluster IV had three different polymorphisms at either *gyrA* (A90V or D94N) or *rrs* (A1401G) locus. Cluster V comprised 3 isolates, the XDR had *gyrA* (A90V or D94G) and *rrs* (A1401G) mutations sharing with 2 isolates, whereas one Pre-XDR isolate with *gyrA* (D94G) and *eis* promoter C (−14) T. Cluster VI had one XDR isolate with a mutation at *rrs* (A1401G) and FQ with a mutation at *gyrA* (D94G).

## Discussion

To our best knowledge, the present study is the first to provide a description of mutations associated with SLD^r^ in *M. tuberculosis* strains from Hebei. Nevertheless, we examined the phylogenetic diversity in SLD^r^ strains through genotyping profiles. The present findings revealed that 48.2% (124/257) were resistant to at least one five SLD. Previously, several studies reported the resistance rate of SLD strains has been estimated to range from 25.7 to 51.9% (Bakuła et al., 2016; Brossier et al., 2017; Hu et al., 2017). According to a recently published World Health Organization (WHO) report, among cases of MDR-TB in 2017, 8.5% (*95%CI*, 6.2–11.0%) were estimated to have XDR-TB (World Health Organization [WHO], 2018). We reported the frequency of XDR-TB is 10.9%.

FQ^r^ isolates is primarily attributed to mutations in the QRDR of *gyrA*. According to a systematic review, *gyrA* mutations were reported in codons 88–94 appeared to account for roughly 60.0–90.0% of FQ^r^ globally (Avalos et al., 2015). In our present study, mutations at *gyrA* codon 89, 90, 91, and 94 were observed in 75.3% of resistant isolates. The frequency of mutations conferring FQ^r^ is similar to Shanghai (76.0%), although it is lower than those in Russia (83.0%), India (81.0%) and Thailand (92.3%), and higher than those in Morocco (30.0%) and New York (67.0%) (Sullivan et al., 1895; Mokrousov et al., 2008; Zhu et al., 2012; Disratthakit et al., 2016; Singhal et al., 2016; Chaoui et al., 2018), indicating that mutations in *gyrA* tend to differ by geographic region. In line with previous studies (Singhal et al., 2016; Pang et al., 2017; Chawla et al., 2018), substitutions at codon 94 were the most frequent mutation among FQ^r^ isolates. This phenomenon may be explained by the fact that codon 94, which aims at the water-magnesium ion bridge with a conserved C3/C4 keto acid moiety of quinolones, plays an important role in stabilizing the quinolone molecule in the quinolone binding pocket, an amino acid substitution at this position will exaggerate the deleterious effect of the binding between most quinolones and DNA gyrase (Aldred et al., 2016; Blower et al., 2016; Disratthakit et al., 2016).

Interestingly, significant evidence has demonstrated a link between *gyrA* mutations and FQ, MDR, Pre-XDR and XDR, suggesting that mutations at *gyrA* might be act as a candidate diagnostic marker for FQ, MDR and a possible indicator of Pre-XDR-TB or XDR-TB. In *Mycobacterium*, the interactive effect between rifampicin- and FQ-resistant mutations was influenced by epistasis and produced a varying degree of loss in fitness (Borrell et al., 2013). We hypothesized that the progression of MDR to Pre-XDR or XDR may be caused by the positive epistasis between *gyrA* mutations and mutations in drug resistant gene conferring rifampicin. Certainly, further research is required to confirm this hypothesis.

Unlike the high frequency of *gyrA* mutations, mutations in *gyrB* gene are seldom commonly associated with FQ^r^ in *M. tuberculosis* isolates. A *gyrB* mutation (L442L, S447F, N499T, A504V, or A508A) was identified in 7 FQ^r^ isolates. As far as we known, a L442L, S447F, N499T, A504V mutation has not been previously reported. Due to the small numbers of isolates with *gyrB* mutations, no such mutation was significant independently associated with FQ^r^. Additionally, we found that both FQ^r^ and FQ^s^ strains exhibited an A508A mutation in *gyrB*, making it difficult to acknowledge the contribution to phenotypic resistance. Several publications reported double mutations in *gyrA*, *gyrB* or both *gyrA* and *gyrB*, which ranged from 1.0 to 3.0% (Avalos et al., 2015; Chien et al., 2016; Kateete et al., 2019). Double mutations in both *gyrA* and *gyrB* were found in FQ^r^ isolates but the absence of susceptible isolates, suggesting that although rare, it can be as the highly specific predictor of FQ^r^.

Previous reports indicated the A1401G in *rrs* effectively identify the phenotypic resistance to KAN, AMK and CAP (Malinga et al., 2016; Varghese and Al-Hajoj, 2017). In our study, 36.5, 63.2, and 50.0% of sensitivities with an A1401G mutation in *rrs* were resistant to KAN, AMK, and CAP, respectively (Supplementary Tables S6–S8). Compared to DST test, it seems that a mutation at A1401G provided a better maker for AMK and CAP than for KAN. However, the canonical A1401G *rrs* mutation explains only around 56.0% of KAN^r^, while the mutations G10A and C14T in the promoter of *eis*, explain another 33.0% of low-level KAN^r^ as estimated by one review (Georghiou et al., 2012). Our study supports these finding, as 5 KAN^r^ isolates were found to have mutations in *eis* promoter, including three of G (−10) A and two of C (−14) T. Mutations in *tlyA* is also known to responsible for resistance to CAP. In fact, we observed three isolates were resistance to CAP alone, but KAN-sensitive and AMK-sensitive isolates had no mutations in *tlyA* gene. Consequently, *tlyA* should be included in the molecular analysis of CAP-resistance.

In our study, *rrs* mutations were significantly associated with MDR-TB (5.1 *OR*, *95%CI* [2.0, 13.0], *P* < 0.001), and XDR-TB (24.1 *OR*, *95%CI* [9.4, 62.2], *P* < 0.001), suggesting the detection of *rrs* mutations provide the valuable information to support the initiation of effective treatment regimens for MDR-TB or even XDR-TB cases. Specifically, as previously reported (Zhang et al., 2014; Ogari et al., 2019), we observed a significant cross-resistance between KAN, AMK and CAP associated with *rrs* gene. Therefore, *rrs* might serve as a marker to predict the cross-resistance among SLID agents.

The traditional phenotypic DSTs lead serious delays to the detection of resistance just because of the extremely slow growth of *M. tuberculosis*. However, several commercial diagnostic tests such as GeneXpert MTB/RIF, Genotype MTBDRplus/MTBDRsl assays, have been developed to rapidly detect both first- and second-line drug resistance in *M. tuberculosis* by scanning the associated mutations (Xie et al., 2017; Kim et al., 2019). Our performance showed that analysis of the associated mutations was recommended to provide a good sensitivity for rapid verification of FQ^r^ (79.4%), SLID^r^ (50.8%), Pre-XDR (80.4%), and XDR (92.9%). In addition, sensitivities and specificities for predicting phenotypic resistance to five SLD in *M. tuberculosis* isolates as shown in Supplementary Tables S4–S8.

Spoligotyping result showed that the predominance of Beijing family (SIT1), which accounted for the largest cluster (71.8%) among SLD^r^ strains in this study. No significant associations were found between the genotypes and specific types of resistance. But a high frequency of Beijing family strains among FQ^r^ isolates was previously reported from Vietnam and Russia (Mokrousov et al., 2008; Duong et al., 2009). One possibility is that the Beijing family strains have a high level of intrinsic resistance to FQ, providing an opportunity to tolerate low level of FQ and subsequently generate *gyrA* mutations, or that these mutations confer an advantage under the absence of antibiotics pressure (Mokrousov et al., 2008). An observation revealed that *eis* mutations were considered to be associated with the Beijing clades (Casali et al., 2014); however, this is not applicable for all geographical settings. Generally, we found no significant correlation between any mutation and a spoligotype family, which is accordance with reports from China (Zhang et al., 2013, 2014).

In this study, an UPGMA-tree was generated from 15-loci MIRU-VNTR among 124 SLD^r^ isolates, which was slightly similar with our previous study on the acquired resistance of MDR-TB in this region (Li et al., 2019). The highly different genotypic patterns and drug resistance profiles suggest that acquisition of resistance is also an important cause for the emergence of SLD^r^ in Hebei Province. MIRU-VNTR data revealed a moderate level of genotypic diversity, which implicated composite mechanisms of resistance, including transmitted and acquired resistance, as a potential cause for the emergence of SLD^r^ strains. Transmission of clustered SLD^r^ strains with identical mutations may indicated the acquisition of drug resistance typically confers a reduction in fitness cost, and mutations may further contribute to the spreading of Pre-XDR-TB or XDR-TB. Therefore, the strict DOTS is the necessary principle to reduce the incidence of acquired strains. In addition, 28.6% of XDR strains were clustered, and it can be inferred that there exists cloning transmission between a small number of XDR strains.

The main limitation of this study was only ability to perform at critical concentrations for DST but not established MICs. This discrepancy of the critical-concentration method used for DST, such that up to 5% of wild-type *M. tuberculosis* strains are classified as drug resistant. While our study is also limited by the number of strains, further studies continue to collect larger additional strains to depict acquired resistance in the community and household and build transmission chain.

## Conclusion

In summary, this study showed that 48.2% of isolates were resistance to at least one of five SLD, and most of which were resistant to OFX, LVX, and KAN. The majority of FQ^r^ and SLID^r^ strains were associated with *gyrA* mutation at D94G and *rrs* mutation at A1401G, respectively. Mutations in *gyrA*, *rrs*, and *eis* promoter seem to be the causative biomarker for the screening of resistance to SLD, even XDR-TB itself. No correlation was found between any mutation and a spoligotype family. Furthermore, acquired resistance is one of the critical factors driving the SLD^r^ strains in Hebei Province. These results highlight the use of appropriate treatment regimens and the development of early rapid diagnosis are the effective manners for the control of SLD-TB or XDR-TB patients.

## Data Availability

All the data analyzed throughout this research are included in this published article.

## Ethics Statement

Approval for this study was obtained from the Medical Ethics Committee of Scientific Research Project of the Fifth Hospital of Shijiazhuang. Written informed consent was obtained from each participant according to the Federal and Institutional Guidelines.

## Author Contributions

QL performed the DST and DNA extraction, analyzed the data, and wrote the first draft of the manuscript. HG, ZZ, and YT performed the DST and spoligotyping. TL performed the MIRU-VNTR typing. YW collected the samples and supported the study. JL and YL contributed to the conception and design of the study. ED designed the study, collected the samples, and revised the manuscript. All authors read and approved the final manuscript.

## Conflict of Interest Statement

The authors declare that the research was conducted in the absence of any commercial or financial relationships that could be construed as a potential conflict of interest.

## Footnotes

1 http://www.ncbi.nlm.nih.gov/BLAST

2 http://www.pasteur-guadeloupe.fr:8081/SITVITDemo/index.jsp

3 http://www.miru-vntrplus.org

## Abbreviations

AMK: amikacin
CAP: capreomycin
CI: confidence interval
DOTS: directly observed treatment short course
DR-TB: drug-resistant tuberculosis
DST: drug susceptibility testing
FQ: fluoroquinolones
KAN: kanamycin
L-J: Lowenstein-Jensen
LVX: levofloxacin
MDR-TB: multidrug-resistant tuberculosis
MIC: minimum inhibitory concentration
MIRU-VNTR: mycobacterial interspersed repetitive unit–variable number of tandem repeats
*M. tuberculosis*: *Mycobacterium tuberculosis*
NPV: negative predictive value
OFX: ofloxacin
OR: odd ratio
PCR: polymerase chain reaction
PPV: positive predictive value
Pre-XDR: pre-extensively drug-resistant tuberculosis
QRDR: fluoroquinolone resistance-determining region
SIT: spoligotyping international type
SLD: second-line anti-tuberculosis drugs
SLID: second-line injectable drugs
SPSS: statistical package for the social sciences
TB: tuberculosis
XDR-TB: extensively drug-resistant tuberculosis
UPGMA: unweighted pair group method with arithmetic mean
WHO: world health organization.
