# Supplementary material for: Mutation and Transmission Profiles of Second-Line Drug Resistance in Clinical Isolates of Drug-Resistant Mycobacterium tuberculosis From Hebei Province, China
Source: Front Microbiol. 2019 Aug 7;10:1838. doi: 10.3389/fmicb.2019.01838 (PMC6692474; doi:10.3389/fmicb.2019.01838)
Supplement: Supplementary file 1 [file Data_Sheet_1.docx]

**Table S1**

PCR and DNA sequencing primers

| Gene | Sequence (5’-3’) | Position | Product (bp) |
| --- | --- | --- | --- |
| *gyrA*-F | TCGACTATGCGATGAGCGTG | 7387-7871 | 485 |
| *gyrA*-R | CGGGATATTGGTTGCCATGC |  |  |
| *gyrB*-F | GCGCTGACGTCGGTGGTGAA | 6206-6977 | 772 |
| *gyrB*-R | ATTCCGGGTCACTGCGCTGC |  |  |
| *rrs*-F | TCGTGTCGTGAGATGTTGGG | 1472152-1472875 | 724 |
| *rrs*-R | TGCTCGCAACCACTATCCAG |  |  |
| *eis*-F | GCGTAACGTCACGGCGAAATT | 2714911-2715477 | 567 |
| *eis*-R | GTCAGCTCATGCAAGGTG |  |  |
| *tlyA*-F | CATCGCACGTCGTCTTTC | 1917863-1918666 | 804 |
| *tlyA*-R | ACCACAGGAAGTACTCGACA |  |  |

**Table S2**

Drug resistant profiles of 257 clinical drug-resistance *M. tuberculosis* isolates of different genotypes

| Drug ^a^ | No. of isolates | | *P*-value |
| --- | --- | --- | --- |
|  | Beijing (n=235) | Non-Beijing (n=22) |  |
| Fluoroquinolones | 92 | 5 | 0.169 |
| Second-line injectable drugs | 55 | 8 | 0.197 |
| Pre-XDR | 42 | 4 | >0.999 |
| XDR | 28 | 0 | 0.145 |

^a^ Pre-XDR, pre-extensively drug-resistant; XDR, extensively drug-resistant

**Table S3**

Distribution of drug-resistance associated with mutations among Beijing and non-Beijing genotypes

| Drug | Mutation | | No. of isolates | | *P*-value |
| --- | --- | --- | --- | --- | --- |
|  |  |  | Beijing (n=235) | Non-Beijing (n=22) |  |
| Fluoroquinolones | *gyrA* | A90V | 23 | 0 | 0.235 |
|  |  | D94G | 23 | 1 | 0.704 |
|  |  | D94A | 11 | 0 | 0.606 |
|  |  | D94N | 9 | 2 | 0.240 |
|  |  | D94Y | 7 | 0 | >0.999 |
|  |  | D89N | 1 | 0 | >0.999 |
|  |  | S91P | 2 | 0 | >0.999 |
|  |  | R106R | 0 | 1 | 0.086 |
|  | *gyrB* | A508A | 6 | 0 | >0.999 |
|  |  | K441R | 1 | 0 | >0.999 |
|  |  | D461N | 1 | 0 | >0.999 |
|  |  | A504V | 1 | 0 | >0.999 |
|  |  | S447F | 1 | 1 | 0.164 |
|  |  | S447Y | 0 | 1 | 0.086 |
|  | *gyrA*/*gyrB* | A90V/N499T | 1 | 0 | >0.999 |
|  |  | D94G/L442L | 1 | 0 | >0.999 |
|  |  | D94Y/A508A | 1 | 0 | >0.999 |
|  |  | No mutation | 146 | 16 | 0.366 |
| Second-line injectable drugs | *rrs* | A1401G | 19 | 2 | 0.697 |
|  |  | C1483T | 1 | 0 | >0.999 |
|  |  | T1491C | 1 | 0 | >0.999 |
|  |  | A1499G | 1 | 0 | >0.999 |
|  |  | G1454A | 1 | 0 | >0.999 |
|  |  | C1209T | 1 | 0 | >0.999 |
|  |  | A1128G | 1 | 1 | 0.164 |
|  | *eis* promoter | G (-10) A | 3 | 0 | >0.999 |
|  |  | C (-14) T | 2 | 0 | >0.999 |
|  |  | G (-37) T | 1 | 0 | >0.999 |
|  | *tlyA* | K69E | 1 | 0 | >0.999 |
|  |  | A119E | 1 | 0 | >0.999 |
|  |  | K189N | 1 | 0 | >0.999 |
|  |  | No mutation | 201 | 19 | >0.999 |

**Table S4**

Sensitivities and specificities for predicting phenotypic resistance to ofloxacin in *M. tuberculosis* isolates

| Locus | No. of OFX^r^ isolates with mutation (n=84) ^a^ | No. of OFX^s^ isolates with mutation (n=173) | Sensitivity  (%[*95%CI*]) a | Specificity  (%[*95%CI*]) |
| --- | --- | --- | --- | --- |
| *gyrA* only |  |  |  |  |
| D89N | 1 | 0 | 1.2(0.0,7.4) | 100.0(97.3,100.0) |
| E90V | 16 | 7 | 19.0(11.6,29.4) | 96.0(91.5,98.2) |
| S91P | 0 | 2 | 0.0(0.0,5.4) | 98.8(95.4,99.8) |
| D94G | 20 | 4 | 23.8 (15.5,34.6) | 97.7(93.8,99.3) |
| D94A | 6 | 5 | 7.1(2.9,15.5) | 97.1(93.0,98.9) |
| D94Y | 5 | 2 | 6.0(2.2,14.0) | 98.8(95.4,99.8) |
| D94N | 11 | 0 | 13.1(7,22.6) | 100.0(97.3,100.0) |
| R106R | 0 | 1 | 0.0(0.0,5.4) | 99.4(96.3,99.9) |
| *gyrB* only |  |  |  |  |
| K441R | 0 | 1 | 0.0(0.0,5.4) | 99.4(96.3,99.9) |
| S447F | 1 | 0 | 1.2(0.0,7.4) | 100.0(97.3,100.0) |
| S447Y | 0 | 1 | 0.0(0.0,5.4) | 99.4(96.3,99.9) |
| D461N | 1 | 0 | 1.2(0.0,7.4) | 100.0(97.3,100.0) |
| A504V | 1 | 0 | 1.2(0.0,7.4) | 100.0(97.3,100.0) |
| A508A | 1 | 5 | 1.2(0.0,7.4) | 97.1(93.0,98.9) |
| *gyrA* and *gyrB* |  |  |  |  |
| D94G/L442L | 1 | 0 | 1.2(0.0,7.4) | 100.0(97.3,100.0) |
| E90V/N499T | 1 | 0 | 1.2(0.0,7.4) | 100.0(97.3,100.0) |
| D94Y/A508A | 1 | 0 | 1.2(0.0,7.4) | 100.0(97.3,100.0) |
| *gyrA* and/or *gyrB* | 66 | 28 | 78.6(68.0,86.5) | 83.8(77.3,88.8) |
| No mutations in *gyrA* or *gyrB* | 18 | 145 |  |  |

^a^ OFX, ofloxacin; r, resistant;

^b^ s, sensitive

^c^ CI, confidence interval.

**Table S5**

Sensitivities and specificities for predicting phenotypic resistance to levofloxacin in *M. tuberculosis* isolates

| Locus | No. of LVX^r^ isolates with mutation (n=56) ^a^ | No. of LVX^s^ isolates with mutation (n=201) ^b^ | Sensitivity  (%[*95%CI*]) ^c^ | Specificity  (%[*95%CI*]) |
| --- | --- | --- | --- | --- |
| *gyrA* only |  |  |  |  |
| D89N | 1 | 0 | 1.8(0.0,10.8) | 100.0(97.7,100.0) |
| E90V | 14 | 9 | 25.0(14.8,38.6) | 95.5(91.4,97.8) |
| S91P | 2 | 0 | 3.6(0.0,13.4) | 100.0(97.7,100.0) |
| D94G | 15 | 9 | 0.7(0.1,3.1) | 100.0(97.7,100.0) |
| D94A | 3 | 8 | 5.4(1.4,15.8) | 96.0(92.0,98.1) |
| D94Y | 5 | 2 | 8.9(3.3,20.4) | 99.0(96.1,99.8) |
| D94N | 7 | 4 | 12.5(5.6,24.7) | 98.0(94.7,99.4) |
| R106R | 0 | 1 | 0.0(0.0,8.0) | 99.5(96.8,99.9) |
| *gyrB* only |  |  |  |  |
| K441R | 0 | 1 | 0.0(0.0,8.0) | 99.5(96.8,99.9) |
| S447F | 0 | 1 | 0.0(0.0,8.0) | 99.5(96.8,99.9) |
| S447Y | 0 | 1 | 0.0(0.0,8.0) | 99.5(96.8,99.9) |
| D461N | 1 | 0 | 1.8(0.0,10.8) | 100.0(97.7,100.0) |
| A504V | 0 | 1 | 0.0(0.0,8.0) | 99.5(96.8,99.9) |
| A508A | 0 | 6 | 0.0(0.0,8.0) | 97.0(93.3,98.8) |
| *gyrA* and *gyrB* |  |  |  |  |
| D94G/L442L | 0 | 1 | 0.0(0.0,8.0) | 99.5(96.8,99.9) |
| E90V/N499T | 1 | 0 | 1.8(0.0,10.8) | 100.0(97.7,100.0) |
| D94Y/A508A | 1 | 0 | 1.8(0.0,10.8) | 100.0(97.7,100.0) |
| *gyrA* and/or *gyrB* | 50 | 44 | 89.3(77.4,95.6) | 78.1(71.6,83.5) |
| No mutations in *gyrA* or *gyrB* | 6 | 157 |  |  |

a LVX, levofloxacin; r, resistant

^b^ s, sensitive

^c^ CI, confidence interval

**Table S6**

Sensitivities and specificities for predicting phenotypic resistance to kanamycin in *M. tuberculosis* isolates

| Locus | No. of KAN^r^ isolates with mutation (n=52) ^a^ | No. of KAN^s^ isolates with mutation(n=205) ^b^ | Sensitivity  (%[*95%CI*]) ^c^ | Specificity  (%[*95%CI*]) |
| --- | --- | --- | --- | --- |
| *rrs* only |  |  |  |  |
| A1128G | 0 | 1 | 0.0(0.0,8.6) | 99.5(96.9,99.9) |
| A1138G | 0 | 1 | 0.0(0.0,8.6) | 99.5(96.9,99.9) |
| C1209T | 0 | 1 | 0.0(0.0,8.6) | 99.5(96.9,99.9) |
| A1401G | 18 | 3 | 34.6(22.3,49.2) | 98.5(95.5,99.6) |
| G1454A | 0 | 1 | 0.0(0.0,8.6) | 99.5(96.9,99.9) |
| C1483T | 0 | 1 | 0.0(0.0,8.6) | 99.5(96.9,99.9) |
| T1491C | 1 | 0 | 1.9(0.1,11.6) | 100.0(97.7,100.0) |
| A1499G | 1 | 0 | 1.9(0.1,11.6) | 100.0(97.7,100.0) |
| *eis* promoter only |  |  |  |  |
| G (-10) A | 3 | 0 | 5.8(1.5,16.9) | 100.0(97.7,100.0) |
| C (-14) T | 2 | 0 | 3.8(0.0,14.3) | 100.0(97.7,100.0) |
| G (-37) T | 0 | 1 | 0.0(0.0,8.6) | 99.5(96.9,99.9) |
| *rrs* and *eis* promoter | 25 | 9 | 48.1(34.2,62.2) | 95.6(91.6,97.8) |
| No mutations in *rrs* or *eis* promoter | 27 | 196 |  |  |

^a^ KAN, kanamycin; r, resistant

^b^ s, sensitive

^c^ CI, confidence interval

**Table S7**

Sensitivities and specificities for predicting phenotypic resistance to amikacin in *M. tuberculosis* isolates

| Locus | No. of AMK^r^ isolates with mutation (n=18) ^a^ | No. of AMK^s^ isolates with mutation (n=239) ^b^ | Sensitivity  (%[*95%CI*]) ^c^ | Specificity  (%[*95%CI*]) |
| --- | --- | --- | --- | --- |
| *rrs* only |  |  |  |  |
| A1128G | 0 | 1 | 0.0(0.0,21.9) | 99.5(97.3,99.9) |
| A1138G | 0 | 1 | 0.0(0.0,21.9) | 99.5(97.3,99.9) |
| C1209T | 0 | 1 | 0.0(0.0,21.9) | 99.5(97.3,99.9) |
| A1401G | 11 | 10 | 61.1(36.1,81.7) | 95.8(92.2,97.9) |
| G1454A | 1 | 0 | 5.6(0.2,29.4) | 100.0(98.0,100.0) |
| C1483T | 0 | 1 | 0.0(0.0,21.9) | 99.5(97.3,99.9) |
| T1491C | 0 | 1 | 0.0(0.0,21.9) | 99.5(97.3,99.9) |
| A1499G | 0 | 1 | 0.0(0.0,21.9) | 99.5(97.3,99.9) |
| *eis* promoter only |  |  |  |  |
| G(-10)A | 1 | 2 | 5.6(0.2,29.4) | 99.2(96.7,99.9) |
| C(-14)T | 0 | 2 | 0.0(0.0,21.9) | 99.2(96.7,99.9) |
| G(-37)T | 0 | 1 | 0.0(0.0,21.9) | 99.5(97.3,99.9) |
| *rrs* and *eis* promoter | 13 | 21 | 72.2(46.4,89.3) | 91.2(86.7,93.4) |
| No mutations in *rrs* or *eis* promoter | 5 | 218 |  |  |

^a^ AMK, amikacin; r, resistant

^b^ s, sensitive

^c^ CI, confidence interval

**Table S8**

Sensitivities and specificities for predicting phenotypic resistance to capreomycin in *M. tuberculosis* isolates

| Locus | No. of CAP^r^ isolates with mutation (n=16) | No. of CAP^s^ isolates with mutation (n=241) | Sensitivity  (%[*95%CI*]) | Specificity  (%[*95%CI*]) |
| --- | --- | --- | --- | --- |
| *rrs* only |  |  |  |  |
| A1128G | 0 | 1 | 0.0(0.0,24.1) | 99.6(97.3,99.9) |
| A1138G | 0 | 1 | 0.0(0.0,24.1) | 99.6(97.3,99.9) |
| C1209T | 0 | 1 | 0.0(0.0,24.1) | 99.6(97.3,99.9) |
| A1401G | 8 | 13 |  |  |
| G1454A | 0 | 1 | 0.0(0.0,24.1) | 99.6(97.3,99.9) |
| C1483T | 0 | 1 | 0.0(0.0,24.1) | 99.6(97.3,99.9) |
| T1491C | 0 | 1 | 0.0(0.0,24.1) | 99.6(97.3,99.9) |
| A1499G | 0 | 1 | 0.0(0.0,24.1) | 99.6(97.3,99.9) |
| *eis* promoter only |  |  |  |  |
| G (-10) A | 1 | 2 | 6.3(0.3,32.3) | 99.2(96.7,99.9) |
| C (-14) T | 0 | 2 | 0.0(0.0,24.1) | 99.2(96.7,99.9) |
| G (-37) T | 0 | 1 | 0.0(0.0,24.1) | 99.6(97.3,99.9) |
| *tlyA* only |  |  |  |  |
| K69E | 1 | 0 | 6.3(0.3,32.3) | 100.0(98.0,100.0) |
| A119E | 1 | 0 | 6.3(0.3,32.3) | 100.0(98.0,100.0) |
| K189N | 1 | 0 | 6.3(0.3,32.3) | 100.0(98.0,100.0) |
| *rrs* and *eis* promoter and *tlyA* | 12 | 25 | 75((47.4,91.7) | 89.6(84.9,93.0) |
| No mutations in *rrs* or *eis* promoter or *tlyA* | 4 | 216 |  |  |

^a^ CAP, capreomycin; r, resistant

^b^ s, sensitive

^c^ CI, confidence interval


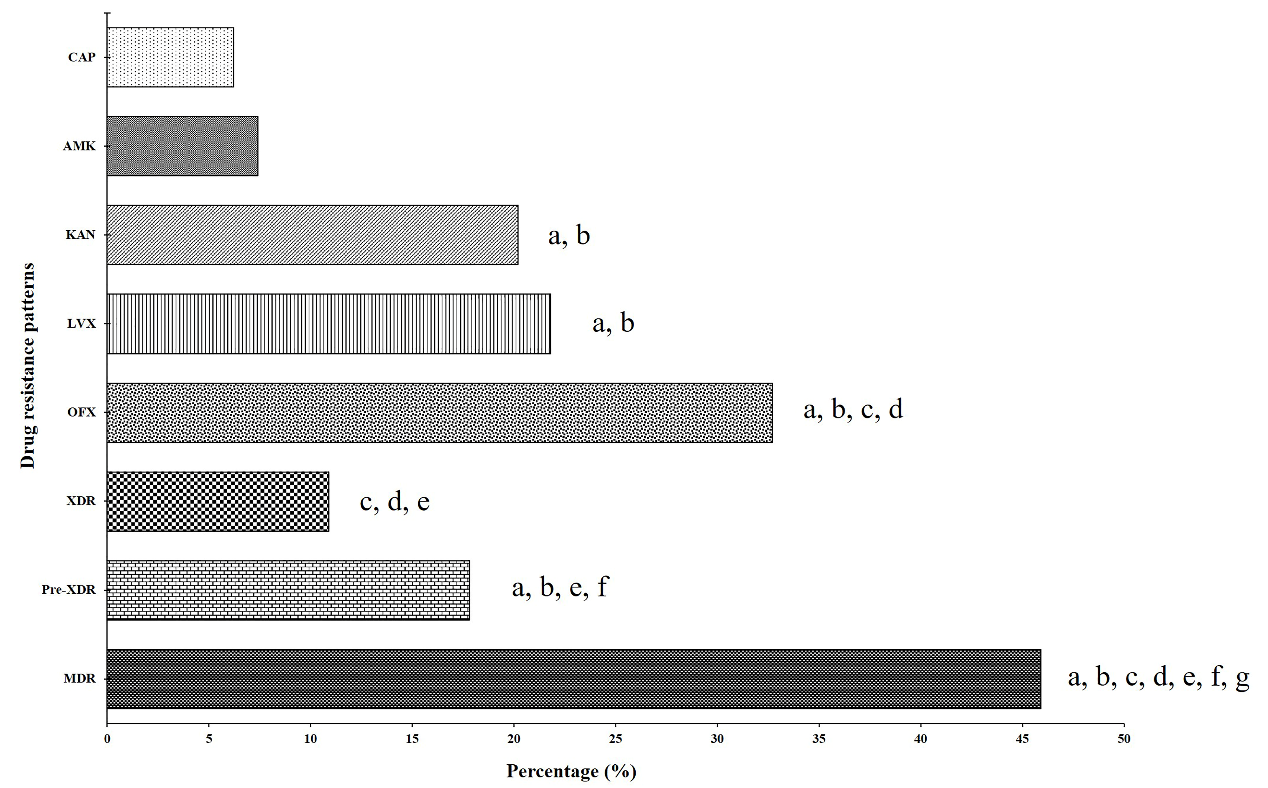


**Fig. S1 Detailed distribution of drug-resistant strains resistant to diﬀerent five second-line drugs group**

^a^ Compared with CAP group, there was a significantly lower than KAN (0.26 *OR*, *95%CI* [0.15, 0.47], *P*<0.001), LVX (0.24 *OR*, *95%CI* [0.13, 0.43], *P*<0.001), OFX (0.14 *OR*, *95%CI* [0.08, 0.24], *P*<0.001), Pre-XDR (0.31 *OR*, *95%CI* [0.17, 0.55], *P*<0.001) and MDR (0.08 *OR*, *95%CI* [0.05, 0.14], *P*<0.001).

^b^ Compared with AMK group, there was significantly lower than KAN (0.32 *OR*, *95%CI* [0.18, 0.55], *P*<0.001), LVX (0.29 *OR*, *95%CI* [0.17, 0.50], *P*<0.001), OFX (0.16 *OR*, *95%CI* [0.09, 0.28], *P*<0.001), Pre-XDR (0.37 *OR*, *95%CI* [0.21, 0.65], *P*<0.001) and MDR (0.09 *OR*, *95%CI* [0.06, 0.16], *P*<0.001).

^c^ Compared with KAN group, there was significantly difference in OFX (0.52 *OR*, *95%CI* [0.35, 0.78], *P*=0.001), XDR (2.75 *OR*, *95%CI* [1.26, 3.41], *P*=0.003) and MDR (0.30 *OR*, *95%CI* [0.20, 0.44], *P*<0.001).

^d^ Compared with LVX group, there was significantly difference in OFX (0.57 *OR*, *95%CI* [0.39, 0.85], *P*=0.006), XDR (2.28 *OR*, *95%CI* [0.39, 3.73], *P*=0.001) and MDR (0.33 *OR*, *95%CI* [0.22, 0.48], *P*<0.001).

^e^ Compared with OFX group, there was significantly difference in XDR (3.97 *OR*, *95%CI* [2.48, 6.36], *P*<0.001), Pre-XDR (2.23 *OR*, *95%CI* [1.48, 3.36], *P*<0.001) and MDR (0.57 *OR*, *95%CI* [0.40, 0.82], *P*=0.002).

^f^ Compared with XDR group, there was significantly lower than Pre-XDR (0.56 *OR*, *95%CI* [0.34, 0.93], *P*=0.024) and MDR (0.14 *OR*, *95%CI* [0.09, 0.23], *P*<0.001)

^g^ Compared with Pre-XDR group, there was significantly lower than MDR (0.26 *OR*, *95%CI* [0.17, 0.38], *P*<0.001).


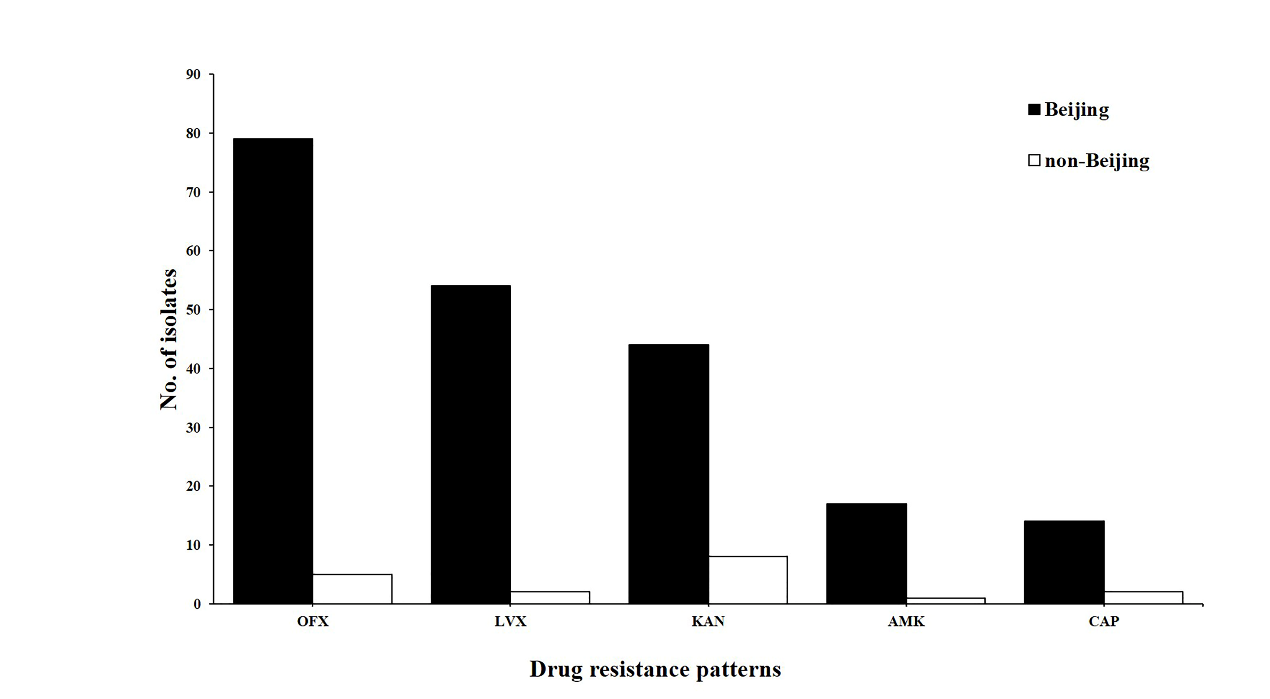


**Fig. S2 the detailed five second-line drugs profiles of 257 clinical drug-resistant *Mycobacterium tuberculosis* isolates of different genotypes**

No significant associations were found between the genotypes and five second-line drug-resistance.
